# Supplementary material for: Molecular spikes: a gold standard for single-cell RNA counting
Source: Nat Methods. 2022 Apr 25;19(5):560–6. doi: 10.1038/s41592-022-01446-x (PMC9119855; doi:10.1038/s41592-022-01446-x)
Supplement: Supplementary file 1 — Reporting Summary [file 41592_2022_1446_MOESM1_ESM.pdf]

nature research

Corresponding author(s): Rickard Sandberg

Last updated by author(s): Mar 04 2022

Reporting Summary

Nature Research wishes to improve the reproducibility of the work that we publish. This form provides structure for consistency and transparency in reporting. For further information on Nature Research policies, see our Editorial Policies and the Editorial Policy Checklist.

Statistics

For all statistical analyses, confirm that the following items are present in the figure legend, table legend, main text, or Methods section.

n/a	Confirmed	
☐	☒	The exact sample size (*n*) for each experimental group/condition, given as a discrete number and unit of measurement
☐	☒	A statement on whether measurements were taken from distinct samples or whether the same sample was measured repeatedly
☒	☐	The statistical test(s) used AND whether they are one- or two-sided<br>*Only common tests should be described solely by name; describe more complex techniques in the Methods section.*
☒	☐	A description of all covariates tested
☒	☐	A description of any assumptions or corrections, such as tests of normality and adjustment for multiple comparisons
☐	☒	A full description of the statistical parameters including central tendency (e.g. means) or other basic estimates (e.g. regression coefficient) AND variation (e.g. standard deviation) or associated estimates of uncertainty (e.g. confidence intervals)
☒	☐	For null hypothesis testing, the test statistic (e.g. F, t, r) with confidence intervals, effect sizes, degrees of freedom and P value noted<br>*Give P values as exact values whenever suitable.*
☒	☐	For Bayesian analysis, information on the choice of priors and Markov chain Monte Carlo settings
☒	☐	For hierarchical and complex designs, identification of the appropriate level for tests and full reporting of outcomes
☒	☐	Estimates of effect sizes (e.g. Cohen's *d*, Pearson's *r*), indicating how they were calculated

Our web collection on statistics for biologists contains articles on many of the points above.

Software and code

Policy information about availability of computer code

Data collection	The following software was used in data collection: FACS sorter software (BD FACSChorus 1.3), sequencer software (MGI DNBSEQ G400RS 1.1.0.108).
Data analysis	Analysis was performed using zUMIs v2.9.5, STAR v2.7.3a, samtools v1.10, pigz v.2.4, R v4.0.4 or v4.1.2, ggplot2 v3.3.3, data.table v1.14.0, Rsamtools v2.6.0, stringdist v0.9.6.3, UMIcountR v0.1.1 (https://github.com/cziegenhain/UMIcountR)

For manuscripts utilizing custom algorithms or software that are central to the research but not yet described in published literature, software must be made available to editors and reviewers. We strongly encourage code deposition in a community repository (e.g. GitHub). See the Nature Research guidelines for submitting code & software for further information.

Data

Policy information about availability of data

All manuscripts must include a data availability statement. This statement should provide the following information, where applicable:

- Accession codes, unique identifiers, or web links for publicly available datasets
- A list of figures that have associated raw data
- A description of any restrictions on data availability

The raw data files for single-cell RNA-sequencing experiments and molecular spikes ground truth sequencing have been deposited in Array Express at European Bioinformatics Institute under accession E-MTAB-10372, E-MTAB-11433 and E-MTAB-11448. Human genome build hg38 fasta files and gene annotation in GTF format (Grch38.95) were obtained from Ensembl.

Field-specific reporting

Please select the one below that is the best fit for your research. If you are not sure, read the appropriate sections before making your selection.

☒ Life sciences ☐ Behavioural & social sciences ☐ Ecological, evolutionary & environmental sciences

For a reference copy of the document with all sections, see nature.com/documents/nr-reporting-summary-flat.pdf

Life sciences study design

All studies must disclose on these points even when the disclosure is negative.

Sample size	A sample-size calculation was not performed before the experiments. Sample size was maximized within reasonable budget for the cost of preparing libraries and sequencing.
Data exclusions	Data filtering steps according to established criteria in single-cell RNA-sequencing were applied only to remove technically failed libraries and described where appropriate. Further data exclusions were not performed.
Replication	For each experimental condition, a large number of single cells was sequenced to ensure reprodocibility. Sample sizes are clearly indicated throughout.
Randomization	Randomization was not performed as the collection of cells (FACS/Dispensing) was done randomly while recording cell size (where possible, as described in the methods section).
Blinding	The researchers were not blinded to the experimental conditions as it was not practically feasible.

Reporting for specific materials, systems and methods

We require information from authors about some types of materials, experimental systems and methods used in many studies. Here, indicate whether each material, system or method listed is relevant to your study. If you are not sure if a list item applies to your research, read the appropriate section before selecting a response.

Materials & experimental systems

n/a	Involved in the study	
☒	☐	Antibodies
☐	☒	Eukaryotic cell lines
☒	☐	Palaeontology and archaeology
☒	☐	Animals and other organisms
☒	☐	Human research participants
☒	☐	Clinical data
☒	☐	Dual use research of concern

Methods

n/a	Involved in the study	
☒	☐	ChIP-seq
☒	☐	Flow cytometry
☒	☐	MRI-based neuroimaging

Eukaryotic cell lines

Policy information about cell lines

Cell line source(s)	HEK293FT cells were purchased from Thermo Fisher.
Authentication	Cell lines were not recently authenticated.
Mycoplasma contamination	Cell lines were confirmed free of mycoplasma contamination using a PCR based test (Eurofins Genomics).
Commonly misidentified lines (See ICLAC register)	No commonly misidentified cell lines were used.
